# Dual isotopic evidence for nitrate sources and active biological transformation in the Northern South China Sea in summer

**DOI:** 10.1371/journal.pone.0209287

**Published:** 2019-01-02

**Authors:** Fajin Chen, Xin Zhou, Qibin Lao, Shuangling Wang, Guangzhe Jin, Chunqing Chen, Qingmei Zhu

**Affiliations:** 1 Guangdong Province Key Laboratory for Coastal Ocean Variation and Disaster Prediction, Guangdong Ocean University, Zhanjiang, China; 2 College of Ocean and Meteorology, Guangdong Ocean University, Zhanjiang, China; 3 Marine Environmental Monitoring Centre of Beihai, State Oceanic Administration, Beihai, China; Chinese Academy of Sciences, CHINA

## Abstract

Nitrate (NO_3_^-^) concentrations and their dual isotopic compositions (δ^15^N-NO_3_^-^ and δ^18^O-NO_3_^-^) were measured to constrain N sources and their cyclic processes in summer using samples from the water column of the northern South China Sea (NSCS). Our data revealed that higher NO_3_^-^ concentrations and δ^15^N-NO_3_^-^ values were observed in the upper waters of the coastal areas near the Pearl River Estuary (PRE). The Bayesian stable isotope mixing model was used to calculated the proportion of nitrate sources, the results indicated that the nitrate in the upper waters of the coastal areas near PRE were mainly influenced by manure and sewage (63%), atmospheric deposition (19%), soil organic nitrogen (12%) and reduced N fertilizer (6%). For the upper waters of the outer areas, low NO_3_^-^ concentrations and δ^15^N-NO_3_^-^ values, but high δ^18^O-NO_3_^-^ values, reflected that NO_3_^-^ was mainly influenced by Kuroshio water intrusion (60%), atmospheric deposition (32%) and nitrogen fixation/nitrification (8%). Complex processes were found in bottom waters. Nitrification and phytoplankton assimilation may be responsible for the higher nitrate concentrations and δ^15^N-NO_3_^-^ values. Our study, therefore, utilizes the nitrate dual isotope to help illustrate the spatial variations in nitrate sources and complex nitrogen cycles in the NSCS.

## Introduction

Nitrogen (N) is a pivotal element in regulating marine primary productivity and plays a prominent role in marine biogeochemistry [1]. Among the various forms of N, nitrate (NO_3_^-^) is the most important form of bioavailable N in the ocean. Recently, anthropogenic activities have greatly increased the input of N to estuaries and adjacent coastal areas and have led to a host of environmental problems, such as eutrophication, hypoxia and harmful algal blooms [2–5]. Therefore, improving our ability to trace N sources, its turnover processes, and dispersal behaviours in estuaries and coastal oceans offers broad benefits.

The South China Sea (SCS) is an oligotrophic marginal sea in the Pacific, and the Pearl River Estuary (PRE) is an interface connecting the Northern SCS (NSCS) and the mainland of South China. Dissolved inorganic nitrogen (DIN) input into the river from the mainland of south China would be finally discharged into the PRE and adjacent coastal areas. On the other hand, the SCS is also frequently influenced by Kuroshio intrusion through the Luzon Strait [6–9]. The Kuroshio Current is characterized by warm and saline surface waters and carries the most oligotrophic water in the world’s oceans into the NSCS [7, 10, 11]. By encountering the warm, saline and oligotrophic from the Kuroshio Current and experiencing the influence of the Pearl River diluted water input, the NSCS becomes a highly complicated and dynamic system. Under such complex hydrodynamic conditions, complex N cycles and sources are present; the spatial NO_3_^-^ distribution controlled by the dynamics of the biogeochemistry is still unclear across the NSCS.

The abundance of ^15^N and ^18^O in nitrate (δ^15^N-NO_3_^-^ and δ^18^O-NO_3_^-^) have proven to be useful in identifying N sources in marine ecosystems [2, 12–16]. For example, NO_3_^-^ originating from inorganic fertilizers is characterized by modest δ^15^N-NO_3_^-^ values (-4 ~ +4‰) [16]. However, NO_3_^-^ originating from sewage and manure is usually enriched in δ^15^N-NO_3_^-^ values (+7 ~ +25‰) due to ammonia volatilization, which may cause enriched δ^15^N in residual nitrate [17, 18]. During the N cyclic processes, depleted δ^15^N-NO_3_^-^ can result from the preferential biological uptake of light N isotopes (^14^N) during nitrification or minimal isotope fractionation during N_2_ fixation [19]. Using the difference in isotopes in nitrate between various sources, Ye et al. [2] suggested that municipal sewage and remineralized soil organic N were the major sources of NO_3_^-^ in the PRE. However, N loss processes, such as assimilation and denitrification, can confound the distinction of NO_3_^-^ sources. The preferential uptake of the lighter isotope in NO_3_^-^ during these processes can lead to ^15^N enriched in the remaining NO_3_^-^ pool [13, 20]. In addition, the proportion of ^18^O in nitrate (δ^18^O-NO_3_^-^) is a powerful tool to distinguish the NO_3_^-^ sourced from atmospheric deposition, which has higher values (>50‰) [21, 22] compared to NO_3_^-^ generated biologically in water and soil (0.8 ~ 5.8‰) [23, 24]. The dual isotope approach can therefore serve as an important tool in many investigations [2, 16, 19, 25] and help deconvolute multiple nitrate sources and biological processes in coastal areas.

In this study, δ^15^N-NO_3_^-^, δ^18^O-NO_3_^-^, NO_3_^-^ and NO_2_^-^ concentrations and other physiochemical parameters were measured in the NSCS during the summer (June 2017). The dual isotope approach enables us to qualitatively characterize the predominant sources of nitrate in coastal areas and understand the controlling factors influencing nitrate distributions.

## Materials and methods

### Field sampling

**Fig 1:**
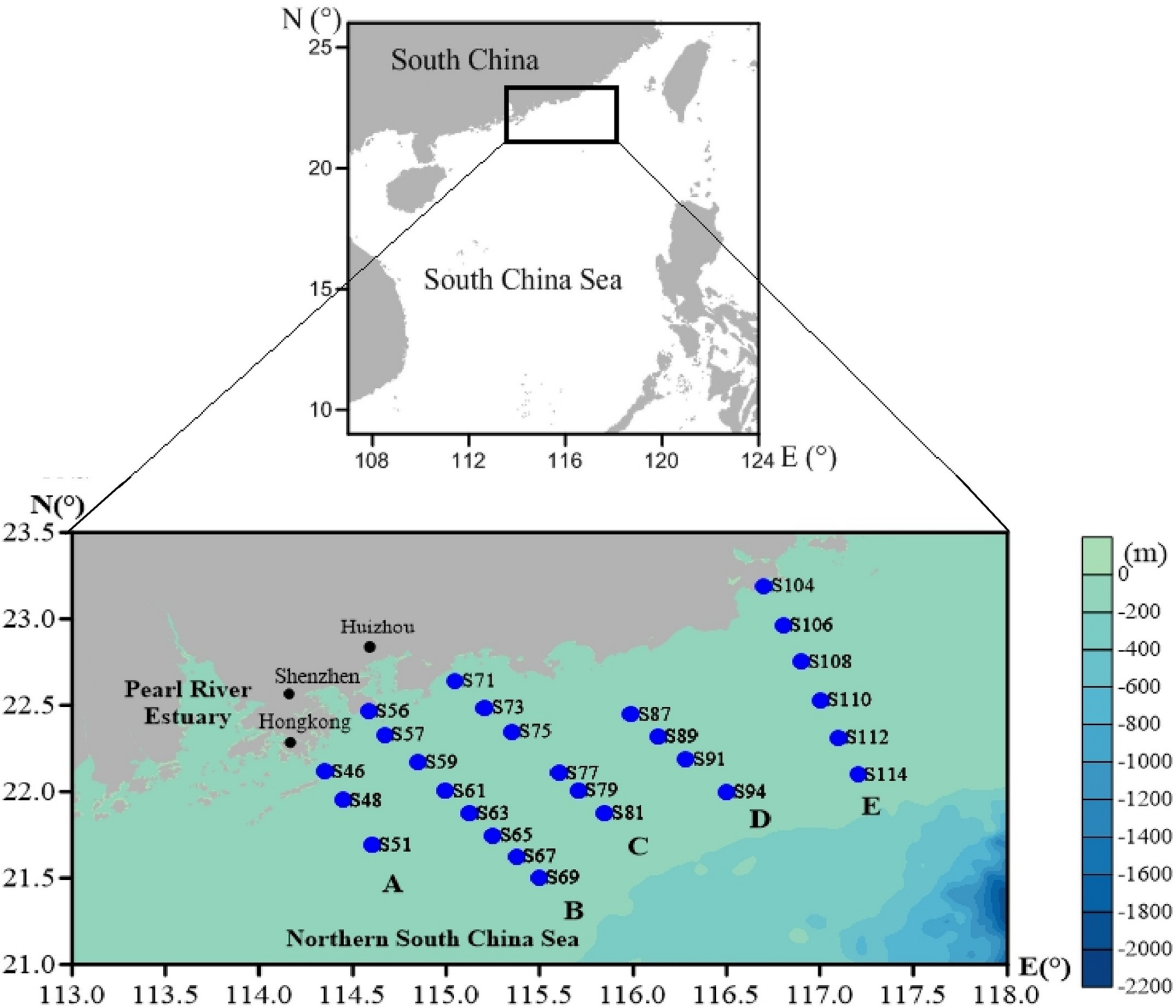
Sampling sites in the NSCS during the summer cruise.

A sampling cruise was carried out in the summer (June) of 2017. Twenty-seven sampling stations were visited in five representative transects (A, B, C, D, and E) to investigate the spatial distribution of NO_3_^-^ and its dual isotopic composition within the NSCS; the sampling sites are presented in Fig 1. No specific permissions were required for the sampling area, because the sampling area did not belong to the nature reserve and not involve endangered or protected species.

At each site, water samples were collected from various depths (2m, 25m, 50m, 75m, 100m, including surface, middle and bottom water) in the water column in 12 L Niskin bottles with a conductivity-temperature-depth (CTD) metre (SBE911, Seabird). Samples for chlorophyll a (Chl-a) were filtered using glass-fibre filters (Whatman, 0.7 μm, GF/F) and stored at -20 °C before analysis. For nutrients and the dual isotopic NO_3_^-^ analysis, water samples of 500 ml were filtered through pre-combustion (450 °C, 4 h) GF/F membranes (47 mm diameter, Whatman), and the filtrate was placed into an acid-washed polyethylene bottle and stored at -20 °C for home lab analysis. This study did not make any damage to the endangered or protected species. This study was carried out without any ethics problem.

### Chemical analyses

The concentration of DO was measured on-site using Winkler titration [26], and the precision was 0.07 mg L^-1^. Chl-a was extracted using 90% acetone and analysed spectrophotometrically [27]. For the nutrients, NO_3_^-^ and NO_2_^-^ were determined using a San++ continuous flow analyser (Skalar, Netherlands). The data quality was monitored by intercalibrations, and the detection limits for NO_2_^-^ and NO_3_^-^ were 0.1 μmol L^-1^. For the dual isotopes of nitrate, nitrite was removed by sulfamic acid, and then isotopic analyses of δ^15^N-NO_3_^-^ and δ^18^O-NO_3_^-^ were conducted following a chemical conversion method, which was modified by Mcllvin and Altabet [28]. According to this method, NO_3_^-^ was reduced to NO_2_^-^ with spongy cadmium and further reduced to N_2_O with sodium azide in an acetic acid buffer. Finally, N_2_O was separated, purified and analysed for N and O isotopes with a GasBench II-MAT 253. δ^15^N and δ^18^O were calibrated by the international standard IAEA-N3. The analysis deviation for the standard was < 0.2‰ for δ^15^N and < 0.5‰ for δ^18^O. The reproducibility of the duplicate sample analyses was < 0.3‰ for δ^15^N (average±0.1‰) and < 0.6‰ for δ^18^O (average±0.3‰).

### Data analysis

Nitrate from the physical mixing (N_mix_) of marine and Pearl River water end-members was calculated for the coastal areas near the PRE using the conservative mixing model of Liss [19], as fllowing:

$$q_{1}+q_{2}=1$$ (1)

$$q_{1}S_{1}+q_{2}S_{2}=S_{mix}$$ (2)

$$q_{1}N_{1}+q_{2}N_{2}=q_{1}N_{mix}$$ (3)

$$q_{1}N_{1}\delta_{1}+q_{2}N_{2}\delta_{2}=N_{mix}\delta_{mix}$$ (4)

where *q*_1_ and *q*_2_ represent the fractional contribution of riverine and marine end-members, respectively. The terms S_1_ and S_2_, N_1_ and N_2_, δ_1_ and δ_2_ represent the parameters of salinity, nitrate and the nitrate dual isotope (δ^15^N-NO_3_^-^ and δ^18^O-NO_3_^-^) of riverine and marine end-members. The terms S_mix_, N_mix_ and δ_mix_ represent the salinity, nitrate and isotopes of a sample in a mixture in the two end-members, respectively. According to the above formula:

$$q_{1}=\frac{S_{mix}-S_{2}}{S_{1}-S_{2}}$$ (5)

$$\delta_{mix}=\frac{\left[q_{1}(\delta_{1}N_{1}-\delta_{2}N_{2})+\delta_{2}N_{2}\right]}{N_{mix}}$$ (6)

Under steady-state conditions, nitrate concentration varies linearly along the mixing gradient, whereas the salinity-based isotopes show curvilinear behaviors, which reflect the concentration-weighted volumes of the two end-members. However, deviations of measured nitrate concentrations or δ^15^N-NO_3_^-^ and δ^18^O-NO_3_^-^ values from the conservative curves may indicate addition of nitrogen from other sources or/and occurrence of nitrogen processing [2]. In this study, the riverine end-member in the upper estuary and the subsurface water from the NSCS were used as the riverine and marine end-member (Table 1), respectively, which were also used by previous studies [2, 19]. Because the subsurface water of the NSCS is less effected than other waters by the addition of variable new nitrogen sources, e.g. nitrogen fixation, phytoplankton assimilation and atmospheric deposition. The common phenomena in the NSCS that wind-induced upper water mixing in winter and off-shore upwelling in summer can provide the subsurface nitrate as the ultimate marine end-member for the coastal surface water [2]. The data of the two end-members (Table 1) was reported in the study of Pearl River Estuary by Ye et al. [2].

**Table 1:** Definitions of marine and riverine end-members.

End-member	Salinity	NO_3_^-^ (μmol L^-1^)	δ^15^N-NO_3_^-^ (‰)	δ^18^O-NO_3_^-^ (‰)
Riverine	3.0	70.4	5.7	1.6
Marine	34.5	5.0	4.0	2.5

### SIAR mixing model

SIAR (stable isotope analysis in R) is a software package to conduct the Bayesian stable isotope mixing model, which is used to calculate the relative proportion of several nitrate sources. In the mixing model, the Bayesian framework is utilized to calculate the probability distribution proportion of nitrate sources. The model framework as following:

$$X_{ij}=\sum_{k=1}^{k}P_{k}(S_{jk}+c_{jk})+\varepsilon_{ij}$$ (7)

$$S_{jk}\sim N(\mu_{jk,}\;\omega_{jk}^{2})$$ (8)

$$C_{jk}\sim N(\lambda_{jk,}\;\tau_{jk}^{2})$$ (9)

$$\varepsilon_{jk}\sim N(0,\;\sigma_{j}^{2})$$ (10)

where *X*_*ij*_ is the isotope values (*j* = 2, δ^15^N-NO_3_^-^ and δ^18^O-NO_3_) of the sample *i* (*i* = 1, 2, 3, …, *N*); *S*_*jk*_ is the isotope value j of the source *k* (*k* = 1, 2, 3, …, *K*) and normally distributed with average *μ*_*jk*_, and standard deviation *ω*_*jk*_; *P*_*k*_ is the proportion of source *k*, which is calculated by the SIAR model; *c*_*jk*_ is the fractionation factor for *j* on source *k*, and *c*_*jk*_ is normally distributed with average λ_*jk*_ and standard deviation *τ*_*jk*_; *ε*_*jk*_ is the residual error of the additional unquantified variation between individual samples, and normally distributed with average 0 and standard deviation *σ*_*j*_. A more detailed description of the model can be found in Moore and Semmens [28], Zhang et al. [29] and Xue et al. [30].

## Results

### Physiochemical parameters

**Fig 2:**
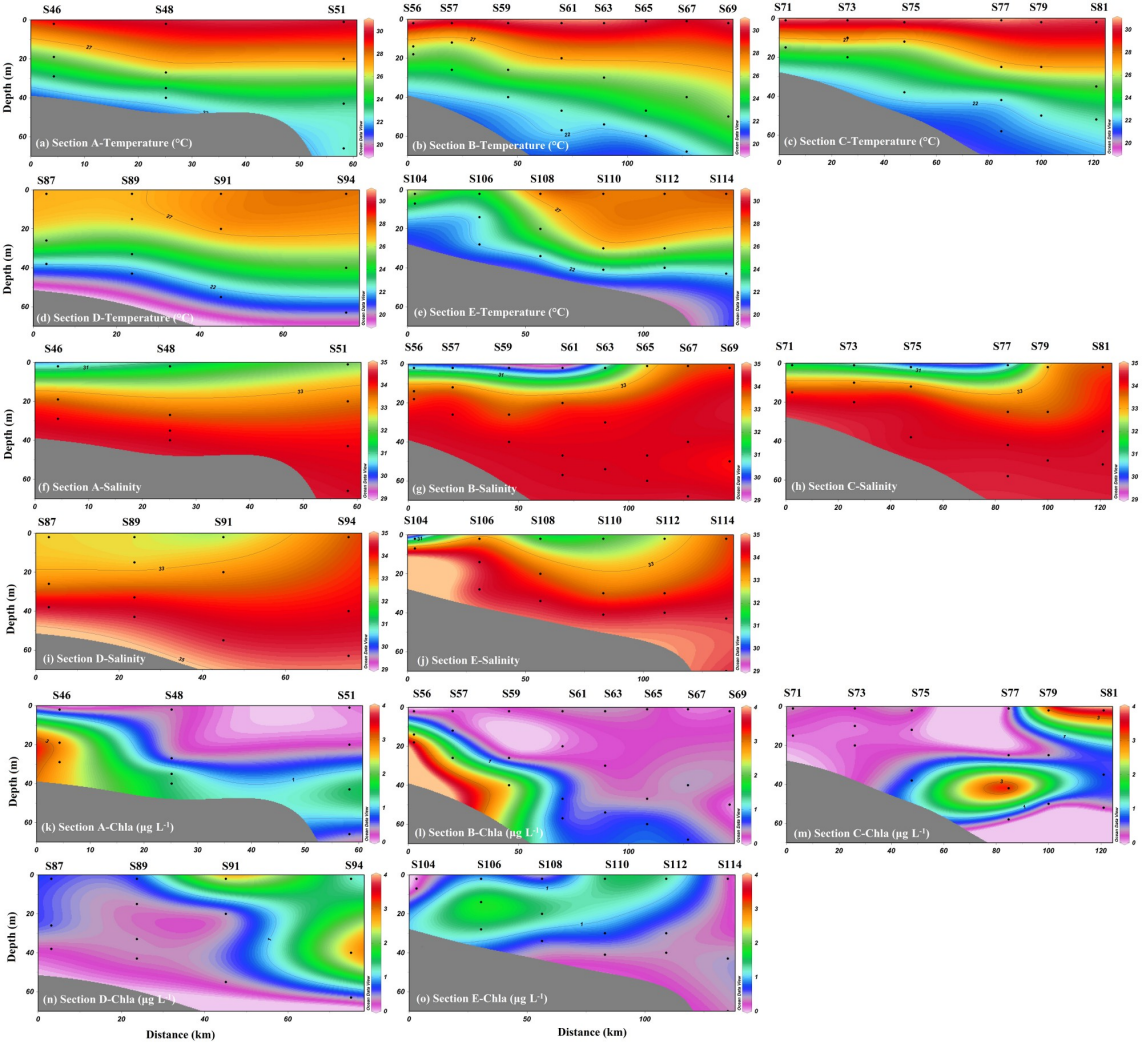
Spatial distributions of temperature, salinity and Chl-a in the NSCS.

The hydrographic characteristics, including the temperature and salinity, exhibited stratified distributions in the NSCS (Fig 2). The temperature and salinity ranged from 19.28 °C to 30.74°C (average of 25.15 °C) and 29.52 to 34.68 (average of 33.46), respectively. Strong thermal stratification was observed in the sampling areas, with a higher temperature in the upper waters and lower temperature in the bottom waters. In contrast, a higher salinity was observed in the bottom waters, whereas a lower salinity was observed in the upper waters of the coastal areas. High temperature and low salinity were observed in the upper waters near the coastal areas of the PRE (Transect A, B and C). However, high temperature (>28.0 °C) and high salinity (33.6) water-column conditions were observed in the outer areas of Transect B, C, D and E. Concentrations of Chl-a ranged from 0.06 to 3.71 μg L^-1^, and higher concentrations were observed in the coastal areas in Transect A and B and the outer areas of Transect C and D.

### Nutrient concentrations

**Fig 3:**
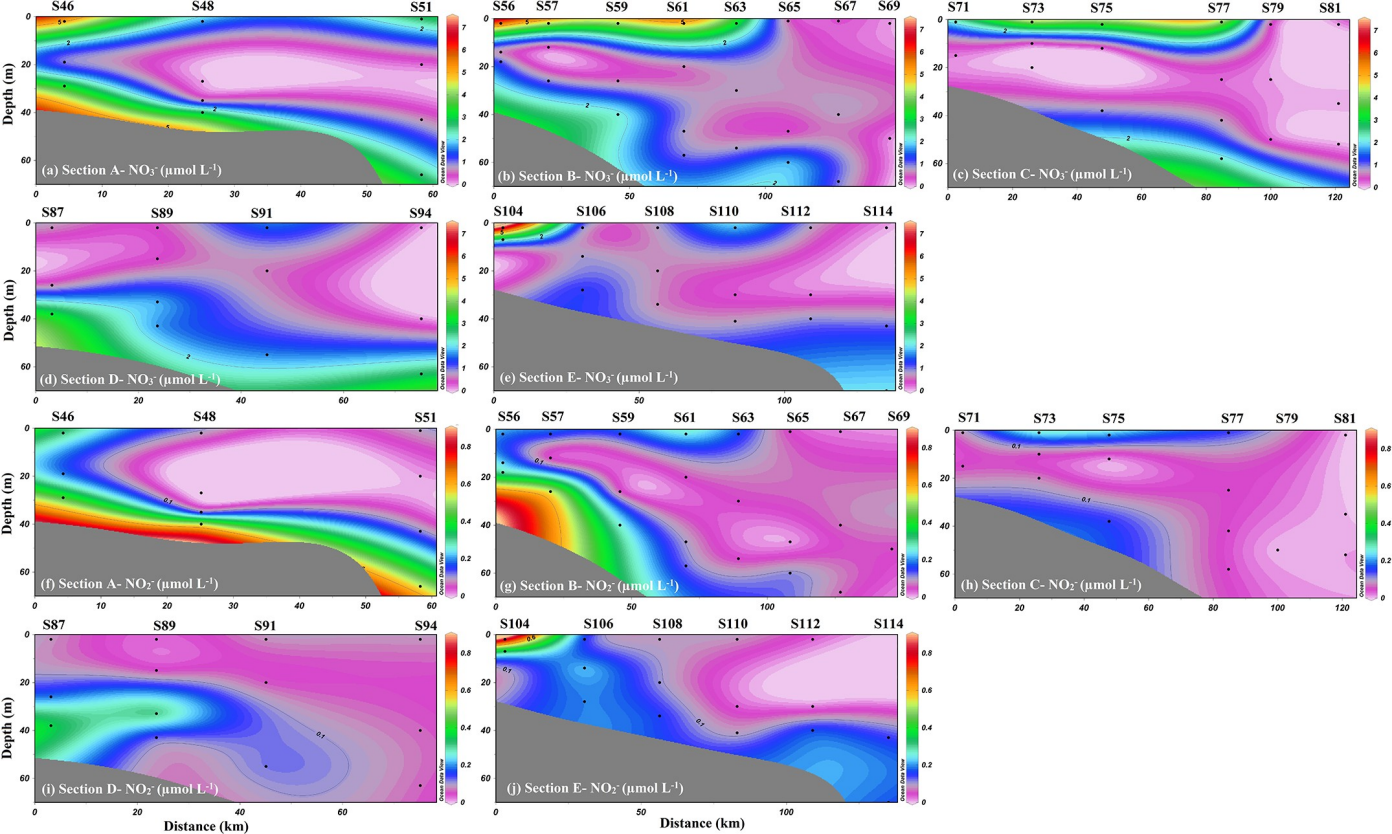
Spatial distributions of NO3- and NO2- in the NSCS. This is the Fig 3 legend.

The concentrations of NO_3_^-^ and NO_2_^-^ ranged from 0.06 to 7.36 μmol L^-1^ (average of 1.50 μmol L^-1^) and ND (no detection) to 0.88 μmol L^-1^ (average of 0.13 μmol L^-1^), respectively. The distribution patterns of NO_2_^-^ were similar to those of NO_3_^-^. As shown in Fig 3, they all exhibited higher levels in the upper water of the nearshore areas in Transect A, B and C. Combined with the distributions of salinity and temperature, these abundant nutrients are probably influenced by riverine input. However, it might not be transported towards the east since low NO_2_^-^ and NO_3_^-^concentrations were observed in Transect D and E. In addition, low concentrations of NO_2_^-^ and NO_3_^-^ were observed in the outer areas and mid-water column. Combined with high temperature and high salinity in the outer areas, this may indicate the influence of the Kuroshio intrusion.

### Isotopic compositions of nitrate

**Fig 4:**
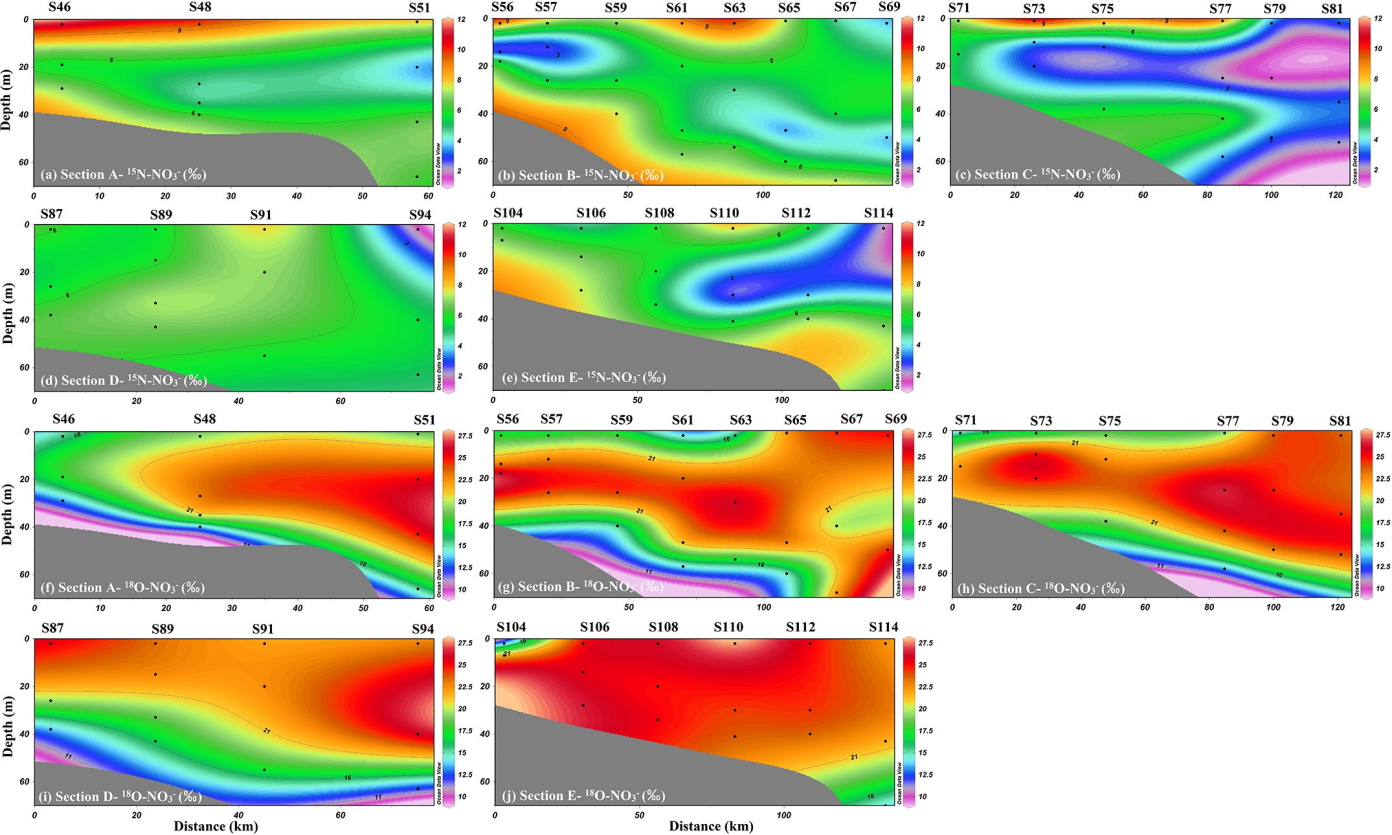
Spatial distributions of δ^15^N-NO3- and δ^18^O-NO3- values in the NSCS.

The isotopic compositions of nitrate, namely δ^15^N-NO_3_^-^ and δ^18^O-NO_3_^-^, ranged in value from 1.49‰ to 11.11‰ and 9.96‰ to 27.86‰, respectively. Similar to the distributions of nutrients, the higher δ^15^N-NO_3_^-^ values were observed in the upper waters near the coastal areas of Transect A, B and C (Fig 4). However, lower values of δ^15^N-NO_3_^-^ were found in the water column of the outer areas in Transect C, D and E. Extremely high values of δ^18^O-NO_3_^-^ were observed in the upper and mid-water column, particularly in the outer areas. The observed high δ^18^O-NO_3_^-^ values (ranged from 9.96‰ to 27.86‰, average of 20.27) in our study areas are at the high end of the range of the δ^18^O-NO_3_^-^ values to be reported in marine environments (-5.0 ~ 33.9‰) [16, 17, 19, 31, 32, 33, 34], and the results are similar to those reported in the lower estuary of the Pearl River (0.7~25.6‰) [2].

## Discussion

### Sources of nitrate in the upper waters of the coastal areas near the PRE

**Fig 5:**
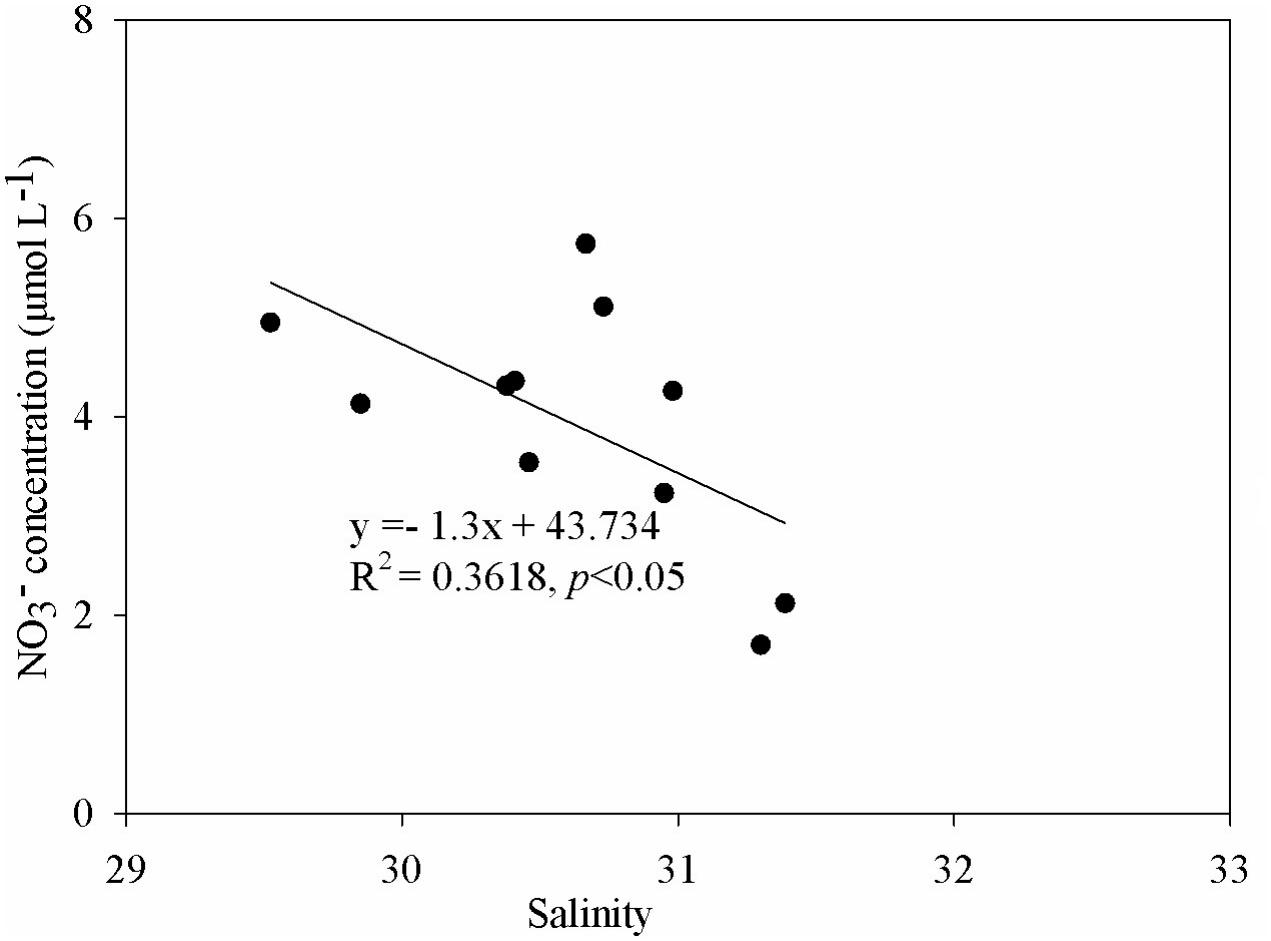
Linear relationship between NO3- concentration, salinity, δ^15^N-NO3- and the natural logarithm of NO3- concentrations in the upper waters near the coastal areas in Transect A, B and C.

As shown in Fig 3, a higher temperature and lower salinity in upper waters near coastal areas in Transect A, B and C (stations S46, S48, S56, S57, S59, S61, S63, S77, S75, S73, and S71) were observed and accompanied by higher NO_3_^-^ concentrations compared to those at other stations. This suggests that the coastal areas near the PRE might be influenced by the Pearl River diluted water and/or the diluted water from the cities adjacent to our study area. This would also be supported by the linear correlation between NO_3_^-^ concentration and salinity (Fig 5). However, the contribution of the Pearl River diluted water can be ruled out, since the Pearl River diluted water near the mouth flows along the west shore due to the Coriolis effect [19]. Because the low temperature and high salinity in the water column were observed in the middle and bottom waters, respectively, the strong thermocline and halocline layer prevented the vertical mixing of water. Thus, the diluted water from the cities adjacent to our study area only influenced the upper waters near the coastal areas in Transect A, B and C.

As shown in Figs 3 and 4, the upper waters of the coastal areas near the PRE are characterized by heavier δ^15^N-NO_3_^-^ and δ^18^O-NO_3_^-^ values (δ^15^N-NO_3_^-^ of 6.78–11.11‰ and δ^18^O-NO_3_^-^ of 14.03–19.42‰) and higher NO_3_^-^ concentrations, which may be associated with biogeochemical processes (such as assimilation and denitrification) and mixing from the upper estuary. However, denitrification are not thought to be responsible for the heavier dual isotopes of nitrate values because high DO level (ranged from 5.01 to 7.07 mg L^-1^, average of 6.09 mg L^-1^) was found. If assimilation was the dominant factor for the heavier δ^15^N-NO_3_^-^ and δ^18^O-NO_3_^-^, NO_3_^-^ would be consumed during the process, leading to low NO_3_^-^ concentrations. Our data are not consistent with this expected pattern. Furthermore, phytoplankton assimilation would be constrained by high turbidity near the coastal areas; a low Chl-a level (0.10–0.34 μg L^-1^) was observed in the coastal areas near the PRE (Fig 2). Thus, higher NO_3_^-^ concentrations and δ^15^N-NO_3_^-^ values would be associated with the diluted water from the cities adjacent to our study area.

**Fig 6:**
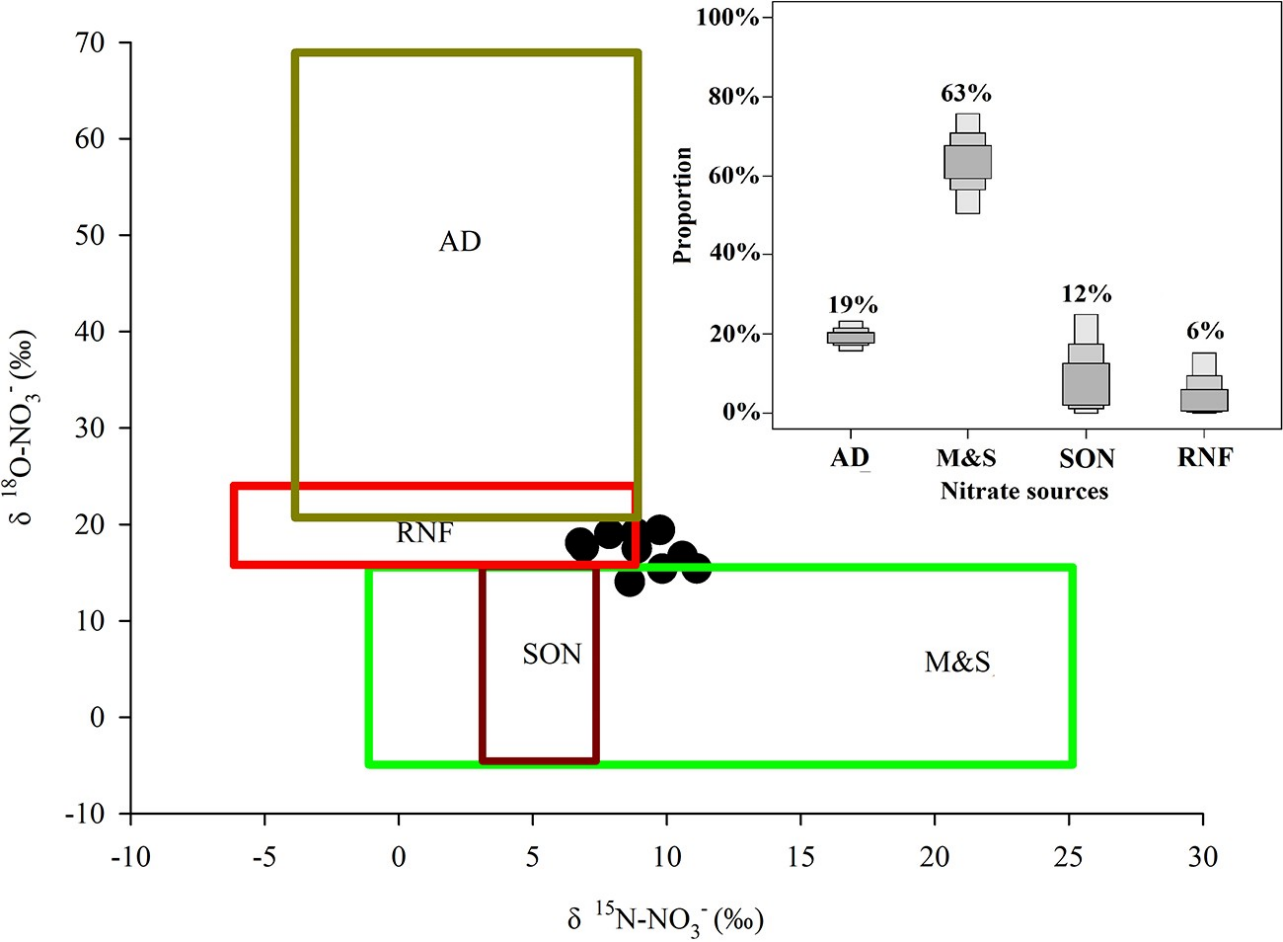
Values of δ^15^N-NO3- and δ^18^O-NO3- in various nitrate sources reservoirs (boxes) [39] and in upper water near the PRE (black dots); the results of the proportion of potential nitrate sources (atmospheric deposition (AD), manure and sewage (M&S), soil organic nitrogen (SON) and reduced N fertilizer (RNF) calculated by Bayesian isotopic mixing model.

From the calculated results of the mixing model, the diluted water from the cities adjacent to our study area in the coastal areas near the PRE comprised 12% of the total, and the subsurface water of the NSCS was 88%. In the diluted water from the cities adjacent to our study area, NO_3_^-^ originated from synthetic NO_3_^-^ fertilizers and atmospheric deposition, and the nitrification of NH_4_^+^ from manure and sewage, reduced N fertilizer, soil organic nitrogen [14, 35]. However, the contribution of synthetic NO_3_^-^ fertilizers can be ruled out, since it accounts for less than 2% of the nitrogen fertilizer applied in China [14]. The slightly higher δ^18^O-NO_3_^-^ values may be influenced by atmospheric deposition and synthetic NO_3_^-^ fertilizers, which have heavier isotopic values (more than 50‰ for atmospheric deposition and +17–+25‰ for synthetic fertilizer) [36, 37]. Since synthetic NO_3_^-^ fertilizers have been ruled out above, atmospheric deposition may be responsible for the slightly higher δ^18^O-NO_3_^-^ values. Previous studies also suggested its importance in providing nitrate for new production in the PRE and the SCS and the higher contribution that was found in the outer-most station [2, 38]. To quantify the contribution of the sources to nitrate in the coastal areas, the Bayesian isotopic mixing model was used in this study. Our results suggest that the fraction of atmospheric deposition (AD) in the coastal areas near the PRE ranged from 16% to 23% (average of 19%), sources from manure and sewage (M&S) ranged from 50% to 76% (average of 63%), soil organic nitrogen (SON) from 0% to 25% (average of 12%) and reduced N fertilizer (RNF) from 0% to 15% (average of 6%) (Fig 6). This result was different from the Pearl River diluted water that the nitrification of reduced N fertilizer was a significant nitrate source, due to most of the land in the Pearl River Basin being used for agriculture [2, 14]. In our study, the cities adjacent to our study area include Hongkong, Shenzhen, Huizhou, which are the most rapid developing cities in China. This would lead that the predominant NO_3_^-^ source in the diluted water from the adjacent cities was manure and sewage. We realize that the uncertainty is large and hard to constrain with the present data. However, such an estimate can at least provide an insight of nitrate sources in the diluted water from the adjacent cities during wet seasons.

### Impact of the Kuroshio intrusion on nitrate in the upper and mid-waters in the outer areas

Except for Transect A, observations from the vertical distributions of the hydrographic characteristics (Fig 2) indicate a high temperature and high salinity in the upper and mid-waters of the outer areas, and the high-salinity water extended towards to the bottom water in the coastal areas, where the salinities were higher than 33.8, even near the coastal areas. These water column conditions are believed to originate from the Kuroshio Current intruding through the Luzon Strait. Similar intrusion patterns have been reported previously [6, 7, 9]. Thus, this phenomenon may introduce low-nutrient waters into the NSCS, during the later intrusion. Our results show significantly low concentrations of NO_3_^-^ and NO_2_^-^ in the water column of the outer areas. Those low-nutrient waters extended to the mid-waters of the coastal areas (Fig 3), which was consistent with the high-salinity distribution patterns. Previous studies also suggested that the Kuroshio intrusion significantly influenced the nutrient distribution in the NSCS and that the nutrient inventory was overall negatively correlated with the fraction of Kuroshio water [7]. However, the distribution patterns of NO_3_^-^ concentration in Transect E were different from those in other transects, which exhibited a higher NO_3_^-^ level along the transects. This nutrient increase may have originated from the Taiwan Strait, which might be less influenced by the Kuroshio Current and exhibit higher NO_3_^-^ concentrations [40].

Interestingly, the higher Chl-a level and lower δ^15^N-NO_3_^-^ values were found in the water column of outer areas in Transect C, D and E (Figs 2 and 3). As mentioned above, oligotrophic water from the Kuroshio intrusion may dilute the nitrate inventory in the water column of the SCS, potentially reducing new production and promoting nitrogen recycling [7, 9]. Nitrification and nitrogen fixation may be responsible for the lower δ^15^N-NO_3_^-^ values in the water column of outer areas. Although the Kuroshio water was characterized by low inorganic nutrients, the dissolved organic nitrogen (DON), dissolved organic carbon (DOC) and total organic carbon (TOC) inventories in the upper 200 m were significantly higher than those in the NSCS [7, 9]. Thus, the high dissolved organic matter (DOM) brought by Kuroshio waters may be bioavailable for microbes, which may activate remineralization and ammonification of DOM [9]. Xu et al., [9] used the ^15^N-NH_4_^+^ to investigated ammonia oxidation (AO) in the NSCS, also indicating that the Kuroshio intrusion enhances NH_4_^+^ regeneration and subsequent oxidation, to complicate conventional new production. Therefore, nitrification would be an important biogeochemical process in outer areas. In addition to nitrification, nitrogen fixation may be another key process in outer areas, as a high abundance of *Trichodesmium* and high N_2_ fixation rates were observed in the path of the Kuroshio Current [41–43]. The high abundance of *Trichodesmium* would stimulate the N fixation in the water column of NSCS, which would increase phytoplankton productivity. The enhancement of nitrogen fixation in the Kuroshio intrusion route would also result in the increase of organic matter mineralization and be further nitrified for the formation of nitrate. In addition, the isotopic feature of δ^15^N-NO_3_^-^ in the Kuroshio Current is approximately 5.6‰ [44, 45], and the value is consistent with the average of the oceanic water over the global ocean (5.0‰) [46]. However, our results from these outer stations (average of 4.9‰) were significantly lower than those of the Kuroshio Current and the average of oceanic water. This further demonstrated that nitrogen fixation and nitrification occurred in the water column of outer areas, which would lead to a lighter isotopic value of nitrate.

However, the extremely high δ^18^O-NO_3_^-^ values were observed in the water column of outer areas, and the middle waters extended into the coastal areas, indicating that it may also be influenced by atmospheric deposition because both nitrification and nitrogen fixation cannot cause the increase in δ^18^O-NO_3_^-^ values. In addition, the mixing with other water masses from the Kuroshio Current and NSCS is not significant enough to lead to δ^18^O-NO_3_^-^ values greater than 10‰ [2, 19]. Since the sampling period was during the rainy season in the NSCS, this would bring high δ^18^O-NO_3_^-^ via atmospheric deposition into the oceanic water. To quantify the contribution of atmospheric nitrate deposition to the water column of the outer NSCS, a simple isotope mass balance based on δ^18^O-NO_3_^-^ was utilized. According to the discussion above, the nitrate sources in the outer NSCS were mainly from nitrogen fixation, nitrification (including the remineralization of organic matter formed by nitrogen fixation), Kuroshio intrusion and atmospheric deposition. The δ^18^O-NO_3_^-^ values of nitrogen fixation, which were further mineralized and nitrified, were calculated to be approximately 7.8‰ [14], and the δ^18^O-NO_3_^-^ values of the Kuroshio Current water were close to the average oceanic water (7.8‰) [41]. Thus, the isotopic feature of nitrogen fixation and nitrification are consistent with the Kuroshio waters, and the input of atmospheric deposition changes the values. To calculate the contribution of atmospheric deposition, we use the following formula:

C=AX+B(1-X) (11)

where C represents the δ^18^O-NO_3_^-^ measured value, A represents the δ^18^O-NO_3_^-^ value for wet deposition (58.8‰)[22], B represents the δ^18^O-NO_3_^-^ value (including nitrification, nitrogen fixation and the Kuroshio Current (7.8‰)), and X represents the contribution rate of wet deposition. Our estimates suggest that the fraction of atmospherically derived nitrate in the outer NSCS ranged from 28%–39% (average of 32%). The nitrate source from atmospheric deposition in the outer areas was higher than that the coastal areas near the PRE (19%) and the PRE (17%) [2], suggesting that the important nitrate sources from atmospheric deposition in the NSCS outside the PRE areas. The results indicated that the contribution of nitrate from nitrification, nitrogen fixation and the Kuroshio Current water was 68%. According to the difference in isotope fingerprint characteristics, we can use δ^15^N-NO_3_^-^ to further calculate the contribution of Kuroshio intrusion, nitrification and nitrogen fixation. The δ^15^N-NO_3_^-^ values of nitrification and nitrogen fixation are close to 0‰, and the value of the Kuroshio Current is approximately 5.6‰ [44, 45]. We use the sample model as follow:

Y=aX+Z(0.647-a) (12)

where Y represents the δ^15^N-NO_3_^-^ measured value, X represents the δ^15^N-NO_3_^-^ value for nitrification and nitrogen fixation (0‰), Z represents the δ^15^N-NO_3_^-^ value of the Kuroshio Current (5.6‰), and a represents the contribution rate of nitrification and nitrogen fixation. The results indicated that the contribution of nitrate sources from nitrification and nitrogen fixation ranged from 0% to 38% (average of 8%) and that from the Kuroshio Current water ranged from 33% to 72% (average of 60%). The proportion of nitrate sources in the outer area was summarized in Table 2. Among the three influencing factors of nitrate concentration, the Kuroshio intrusion was the dominant factor that controlled the nitrate concentration in the outer areas of NSCS. This phenomenon may be responsible for the lower nutrients in the areas.

**Table 2:** The proportion of nitrate sources in the outer area.

Station	Atmospheric deposition	Nitrification/N_2_ fixation	Kuroshio Current
S65	29%	0%	71%
S67	33%	0%	67%
S69	33%	2%	65%
S81	31%	16%	53%
S79	32%	15%	53%
S94	30%	38%	33%
S91	28%	0%	72%
S89	30%	0%	70%
S87	34%	0%	66%
S114	29%	33%	39%
S112	34%	0%	66%
S110	39%	0%	61%
S108	36%	0%	64%
S106	35%	0%	65%
Average	32%	8%	60%

### Complex processes for high nitrate and isotopic values in bottom waters

**Fig 7:**
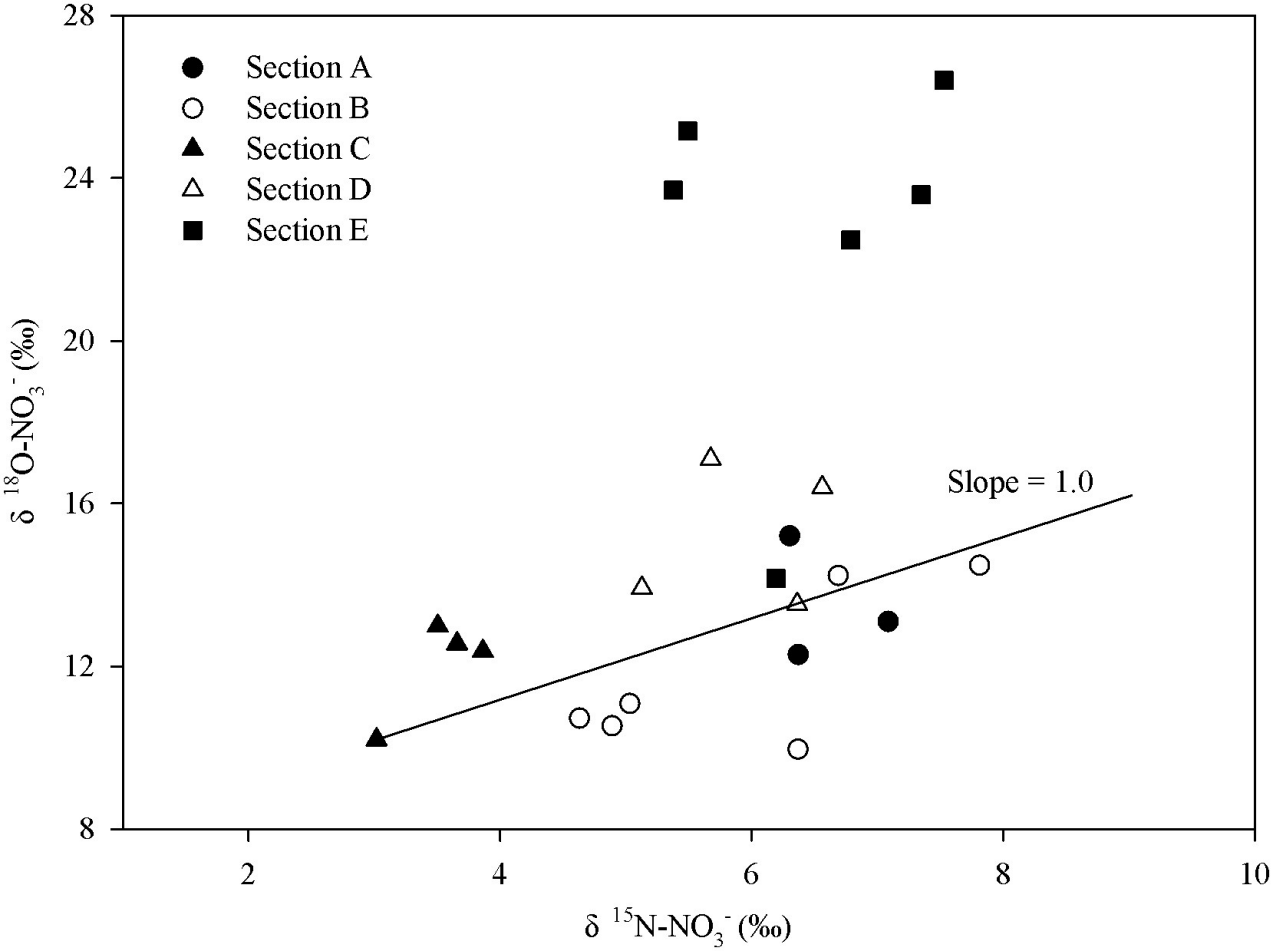
The range of δ^15^N-NO3- and δ^18^O-NO3- values measured in the bottom waters of the NSCS.

In bottom waters, particularly in coastal areas near the PRE (Transect A and B), higher nutrients and δ^15^N-NO_3_^-^ values were also observed, while the δ^18^O-NO_3_^-^ values exhibited lower values compared with the upper waters (Fig 4). Due to the strong thermocline and halocline during the summer, nitrification may be responsible for the higher concentrations of NO_3_^-^ and NO_2_^-^ in bottom waters. However, the higher isotopic values of nitrate observed in the bottom waters indicated that other processes may have also occurred, such as assimilation and denitrification. Denitrification or nitrate assimilation by phytoplankton leads to an increase in δ^15^N-NO_3_^-^ and δ^18^O-NO_3_^-^ values because lighter isotopes ^14^N and ^18^O are preferentially metabolized by microorganisms or phytoplankton. This makes the remaining nitrate pools simultaneously enriched in δ^15^N and δ^18^O at a ratio of 1:1, while nitrification leads to the decoupling of δ^15^N-NO_3_^-^ and δ^18^O-NO_3_^-^ values, as a decrease in δ^18^O-NO_3_^-^ is smaller than that in δ^15^N-NO_3_^-^ [17, 34]. However, denitrification seems unlikely to have occurred due to a relatively high DO level (4.25 ~ 7.03 mg L^-1^, average of 5.41 mg L^-1^) observed in the bottom waters. Thus, phytoplankton assimilation primarily contributed to the enrichment of δ^15^N-NO_3_^-^ and δ^18^O-NO_3_^-^ values in the bottom waters of the NSCS. As shown in Fig 7, except for Transect E, most of the δ^15^N-NO_3_^-^ and δ^18^O-NO_3_^-^ values in the bottom waters fell within the theoretical value (1:1). In addition, an extremely high level of Chl-a was found in the middle and bottom waters of the coastal areas near the PRE (Transect A and B, Fig 2), which further confirmed that phytoplankton assimilation occurred in these areas. However, the δ^15^N-NO_3_^-^ and δ^18^O-NO_3_^-^ values slightly deviated from the assimilation line, which could be influenced by nitrification and other sources. For example, significantly higher δ^18^O-NO_3_^-^ values occurred in Transect E, which may be influenced by atmospheric deposition. Atmospheric deposition would be first deposited in the upper waters of the ocean and then mixed in the water column and transported with ocean currents. In addition, the NH_4_^+^ concentrations (ranged from 0.46 to 3.42 μmol L^-1^, average of 1.18 μmol L^-1^) in the bottom water was significantly lower than NO_3_^-^ concentration (ranged from 0.08 to 4.42 μmol L^-1^, average of 1.94 μmol L^-1^), this further indicated that nitrification indeed occurred at the bottom water.

## Conclusions

Nitrate dual stable isotopes in the water column of the NSCS were investigated to provide information about nitrogen sources and cycles. A higher NO_3_^-^ concentration and δ^15^N-NO_3_^-^ value were observed in the upper waters of the coastal areas near the PRE, which were mainly influenced by manure and sewage (63%), atmospheric deposition (19%), soil organic nitrogen (12%) and reduced N fertilizer (6%). For the upper waters of the outer areas, low NO_3_^-^ concentrations and δ^15^N-NO_3_^-^ values but high δ^18^O-NO_3_^-^ values were observed, which were mainly influenced by Kuroshio intrusion (61%), atmospheric deposition (31%) and nitrogen fixation/nitrification (8%). The results indicated that the important nitrate source from atmospheric deposition in the outer areas. Complex processes were found in the bottom waters, and the nitrification and phytoplankton assimilation may be responsible for the higher nitrate and δ^15^N-NO_3_^-^ values. Our study, therefore, manifests the combination of isotopic and nitrate data to help illustrate the spatial variation in nitrate sources and the complex nitrogen cycles in the NSCS.
